# Antioxidant capacity of cinnamon extract for palm oil stability

**DOI:** 10.1186/s12944-018-0756-y

**Published:** 2018-05-16

**Authors:** Muhammad Zia Shahid, Hafiza Saima, Adeela Yasmin, Muhammad Tahir Nadeem, Muhammad Imran, Muhammad Afzaal

**Affiliations:** 1 Department of Food Science, Nutrition and Home Economics, Government College University, Faisalabad, Pakistan; 2 Institute of Home and Food Sciences, Faculty of Life Sciences, Government College University, Faisalabad, Pakistan

**Keywords:** Cinnamon extract, Antioxidants, Palm oil, Oxidation, Oil stability

## Abstract

**Background:**

Spices and their bioactive components are more promising attractions for their inclusion in diet-based regimes to improve human health. These are sources of natural antioxidants and play an important role in the chemoprevention of diseases and aging. The aim of the current study was to explore the antioxidant potential of cinnamon; a widely used spice throughout the world.

**Methods:**

The current research was aimed to investigate the antioxidant potential of cinnamon extract. For the purpose, cinnamon sticks were procured from local super market, while palm oil was obtained from local oil industry. The resultant extract was analyzed for its antioxidant activity through total phenolic content (TPC), free radical scavenging activity (DPPH assay), and total antioxidant activity was measured by ferric reducing antioxidant power (FRAP) test. The shelf life of palm oil was checked by adding cinnamon extract in oil at different levels _i.e._*,* 0.05, 0.10, 0.15, 0.20 and 0.25%, to compare the antioxidant potential of the extract whereas, T_o_ acted as control and T_BHA_ @ 0.1% was used as synthetic antioxidant in the oil samples. The oil samples were analyzed for rancidity check during storage (after every seven days for a storage period of four weeks).

**Results:**

The results indicated that total phenolic contents (TPC); 1,1-diphenyl-2-picrylhydrazyl (DPPH) and ferric reducing antioxidant power (FRAP) values of cinnamon extract were as 355.01 ± 8.34 gallic acid equivalent per gram (mg GAE/g), 90.18 ± 2.12 (%) and 132.82 ± 3.12 (μmol/g), respectively. The oxidative parameters for treatments i.e.*,* T_o_, T_BHA_, T_1_, T_2_, T_3_, T_4_, T_5_ were recorded as peroxide value (2.61 ± 0.07, 2.42 ± 0.08, 2.57 ± 0.05, 2.56 ± 0.03, 2.54 ± 0.02, 2.54 ± 0.01, 2.46 ± 0.06 meq/kg, respectively), free fatty acids (0.601 ± 0.05, 0.522 ± 0.02, 0.580 ± 0.07, 0.572 ± 0.03, 0.56 ± 00.07, 0.552 ± 0.03, 0.536 ± 0.05%, respectively), TBA value (22.4 ± 1.45, 20.1 ± 0.73, 21.8 ± 0.42, 21.2 ± 1.56, 20.7 ± 0.48, 20.5 ± 0.59, 20.2 ± 0.91 μg/kg, respectively) and iodine value (52.82 ± 2.12, 52.71 ± 2.38, 52.68 ± 2.96, 52.97 ± 2.14, 52.93 ± 2.12, 53.15 ± 2.38, 52.71 ± 2.96, respectively). Overall, the statistical analysis indicated that all parameters regarding oil stability i.e.*,* peroxide value (PV), free fatty acid content, (FFA) thiobarbituric acid (TBA) value and iodine value (IV) were significant with respect to treatments and storage.

**Conclusion:**

From the present study, it can be concluded that the cinnamon extract proved effective in reducing the lipid oxidation of palm oil and it can be successfully used in place of synthetic antioxidants in food preparations.

## Background

Novel health care strategies of the millennium have illuminated functional foods as one of the promising therapeutic tool to curb various lifestyle related maladies. In the recent era health enhancing properties of specific foods have been documented with antioxidant and anti-inflammatory properties [1]. Spices and their bioactive components are more promising attractions for their inclusion in diet-based regimes to improve human health. According to the current nutritional guidelines, diet and health interplay has encouraged the consumers to choose food with some additional health benefits beyond basic nutrition [2]. These are sources of natural antioxidants that play key role in prevention of aging and diseases [3, 4]. Amongst various plants investigated, one showing enormous potential is the cinnamon belonging to family Lauraceae. Cinnamon is gained from the tree bark of cinnamomum genus that is a tropical perennial plant. It has two leading varieties *zeylanicum Cinnamomum* (CZ) and *cassia* (CC) cinnamon [5]*.* The bioactive compounds of essential oils found from CZ bark are trans-cinnamaldehyde, eugenol and linalool, which are representative of almost 82.5% of total compounds [6]. It possesses antimicrobial, antifungal, antioxidant, and antidiabetic activity [7–9]. Moreover, it has also been used as anti-inflammatory, antitermitic, nematicidal, mosquito larvicidal, insecticidal, antimycotic, and anticancer agent [10–12].

Oxidation reactions generates free radicals that start off chain reactions. This spontaneous process of oxidation causes rancidity and food spoilage. Moreover, oxidative stress leads to many lethal diseases in humans like cancer [13]. Currently synthetic antioxidants are used in the food and pharmaceutical industries to extend product shelf life [14]. However, there is increasing interest to explore natural sources of antioxidants to minimize the damage caused by synthetic ones. Phytonutrients are increasingly known for antioxidant activity [15, 16]. Cinnamon spice is one of the sources of effective antioxidants and enhances the efficacy of other important antioxidants. It contains several potent antioxidants and is most important spice used for curtailing oxidative stress. The antioxidant potential of cinnamon is attributed to array of flavonoid compounds that it contains. The essential oils present in cinnamon, including cinnamaldehyde, eugenol, and linalool, has been investigated in reference to peroxynitrite induced nitration and lipid peroxidation [17].

Palm oil is an important part of human and animal diet and plays key role in food processing industry. Its use in the commercial food industry is widespread owing to low cost and frying stability. One of the important quality parameters in oil refining industry is low level of free fatty acid (FFA) content and oxidative products [18]. However most of the times palm oil for domestic use is processed through low grade mills which enhances the FFA content and ultimately leads to oxidation during storage. In order to shield the palm oil from oxidative damage during extended storage and transportation, stabilization of the oil with antioxidants is undoubtedly indispensable to preserve the good oxidative status of the palm oil [19]. The developing countries are spending huge amounts on the import of edible oils from other developed countries and the cinnamon extract can be used to preserve the quality of this product. The present study was aimed to explore the potential of natural antioxidant and its comparison with synthetic one to enhance the storage stability of palm oil. The specific objectives of the current study were as (1) probe the antioxidant potential of cinnamon extract (2) exploration the potential of the natural antioxidants in comparison to synthetic ones, and (3) improving the storage stability of palm oil.

## Methods

### Purchase of raw material

Cinnamon sticks were procured from Metro cash and carry, Faisalabad. They were dried in an oven at 55 °C till the constant moisture content of 8.5%, crushed using electrical grinder, refined & bleached. Deodorized (RBD) palm oil was obtained from United Ghee Industries Ltd., Faisalabad. All chemicals were of analytical grade and were purchased from Sigma-Aldrich (St. Louis, USA); Merck (Darmstadt, Germany) and Fluka (Buchs, Switzerland). The powdered sample were subjected to solvent extraction using ethanol.

### Solvent extraction of bioactive compounds

Solvent extraction was done following the method used by Al-Juhaimi and Ghafoor [20] with modifications using ethanol as solvent. The powdered cinnamon sample (10 g) was extracted (50 mL), for 1 h under agitation at room temperature (25 °C) and was filtered through vacuum filtration assembly. The residues were extracted again in ethanol (50 mL), filtered and stored in a freezer at − 18 °C till for further use.

### Determination of antioxidant potential of the extract

#### Total phenolic content (TPC)

Total phenolic contents of cinnamon extract were determined spectrophotometrically following Folin-Ciocalteu method described previously, [21] with minor modification. The appropriate dilution of extract 200 μl oxidized with 1 mL of Folin-Ciocalteu reagent, and then the reaction mixture was neutralized with saturated 2 mL of 7.5% sodium carbonate (*w*/*v*). The final mixture volume was maintained up to 7 mL with deionized water. After incubation for 2 h in dark at room temperature, absorbance of the resulting blue color was measured at 765 nm using UV-Visible spectrophotometer. Gallic acid was used as a standard for the calibration curve. The phenolic content was determined as gallic acid equivalents using the linear equation based on the calibration curve.

#### Free radical scavenging activity (DPPH assay)

The free radical scavenging activity of the extracts was determined by using 1, 1-diphenyl-2-picrylhydrazyl or DPPH [22]. The scavenging ability *(SA)* was calculated as follows:

$$ \mathrm{SA}\%\kern0.5em =\kern0.5em \frac{\Delta \mathrm{A}\kern0.5em 517\kern0.5em \mathrm{control}\kern0.5em \hbox{-} \kern0.5em \Delta \mathrm{A}\kern0.5em 517\kern0.5em \mathrm{sample}}{\Delta \mathrm{A}\kern0.5em 517\kern0.5em \mathrm{Control}}\kern0.5em \times 100\kern0.5em $$

#### Estimation of total antioxidant activity (FRAP test)

Total antioxidant activity was measured by ferric reducing antioxidant power (FRAP) assay of Benzie and Strain [23].

#### Addition of extract

The shelf life of palm oil was checked by adding cinnamon extract in oil at different levels _i.e._*,* 0.05, 0.10, 0.15, 0.20 and 0.25%, to compare the antioxidant potential of the extract, whereas, T_o_ acted as control and T_BHA_ @ 0.1% was used as synthetic antioxidant in the oil samples.

### Oil stability analysis

To check the antioxidant potential of the extract on the oil stability, following chemical analysis were performed. Peroxide value was measured through procedure No. Cd 8–53 [24]. Free fatty acid content of oil samples was measured by titrating the oil samples dissolved alcohol against sodium hydroxide (NaOH) to a specific end point by procedures described in AOCS method No. Ca 5a-40 [24]. The TBA-reactive substances (TBARS) were determined in oil samples as described by [25]. Iodine value (IV) of the oil samples was determined by [24] method.

### Statistical analysis

All determinations were carried out in triplicate and data was reported as mean ± standard deviation. Data obtained for the different parameters was analyzed using the analysis of variance (ANOVA) technique and the least significance difference (LSD) test was used to compare the means, according to the procedure described by Steel et al. [26].

## Results

### Antioxidant potential of cinnamon extract

The antioxidant activity of cinnamon extract was investigated using total phenolic content (TPC), free radical scavenging activity (DPPH assay), and ferric reducing antioxidant power (FRAP) test and the values were recorded as 355.01 ± 8.34 mg GAE/g, 90.18 ± 2.12 (%) and 132.82 ± 3.12 (μmol/g), respectively.

### Palm oil stability tests

In the current study the mean squares relating to PV, FFA, TBA and IV of palm oil associated with treatments and storage have been discussed in Table 1. After the addition of cinnamon extracts RBD, Palm oil was stored at room temperature for 28 days. The oil stability tests were performed weekly to assess the effectiveness of cinnamon extract against oxidation damage or rancidity. The peroxide value of treatments for palm oil i.e.*,* T_o_, T_BHA_, T_1_, T_2_, T_3_, T_4_, T_5_ was recorded as 2.61 ± 0.07, 2.42 ± 0.08, 2.57 ± 0.05, 2.56 ± 0.03, 2.54 ± 0.02, 2.54 ± 0.01, 2.46 ± 0.06 (Table 2). However, storage caused a significant increase in peroxide value from 0 to 28th day as 2.36 ± 0.03, 2.46 ± 0.08, 2.54 ± 0.06, 2.61 ± 0.04 and 2.68 ± 0.03. The results in the Table 3 revealed that there was a significant difference among treatments on free fatty acid contents of palm oil as the values for T_o_, T_BHA_, T_1_, T_2_, T_3_, T_4_, T_5_ were observed as 0.601 ± 0.05, 0.522 ± 0.02, 0.580 ± 0.07, 0.572 ± 0.03, 0.56 ± 00.07, 0.552 ± 0.03, 0.536 ± 0.05. The free fatty acid content decreased as the concentration of extract was increased. The effect was more pronounced for the synthetic antioxidant BHA, yet cinnamon extract at 0.25% had almost similar effect (Table 3). Similarly, the effect of storage was also significant as the value of free fatty acids increased as the storage period moved from 0 to 28 days. The values for different storage periods i.e., 0, 7, 14, 21 and 28 days were noticed as 0.491 ± 0.02, 0.521 ± 0.05, 0.555 ± 0.07, 0.580 ± 0.06, 0.654 ± 0.06.

**Table 1:** Mean squares for PV, FFA, TBA and IV of palm oil during storage

SOV	df	PV	FFA	TBA	IV
Treatments	6	0.06775^a^	0.01079^a^	10.610^a^	0.4468^a^
Storage (days)	4	0.33322^b^	0.08145^b^	118.649^b^	15.9591^a^
Treatments x storage	24	0.00357^NS^	0.00160^NS^	2.421^NS^	0.1246^NS^
Error	70	0.00445	0.00018	0.313	1.9391

*NS* Non-significant, *PV* peroxide value, *FFA* free fatty acids, *TBA* thiobarbituric acid, *IV* iodine value

^a^Significant

^b^Highly significant

(*p* < 0.05)

**Table 2:** Effect of treatments and storage on peroxide value (meq/kg) of palm oil

Treatments	Storage (days)					Means
	0	7	14	21	28	
T_o_	2.36 ± 0.06	2.55 ± 0.06	2.63 ± 0.08	2.69 ± 0.04	2.83 ± 0.03	2.61 ± 0.07^a^
T_BHA_	2.33 ± 0.03	2.34 ± 0.04	2.41 ± 0.02	2.47 ± 0.06	2.55 ± 0.03	2.42 ± 0.08^d^
T_1_	2.36 ± 0.06	2.51 ± 0.06	2.59 ± 0.03	2.66 ± 0.08	2.73 ± 0.06	2.57 ± 0.05a^b^
T_2_	2.36 ± 0.05	2.50 ± 0.02	2.58 ± 0.06	2.66 ± 0.06	2.71 ± 0.02	2.56 ± 0.03^b^
T_3_	2.37 ± 0.03	2.49 ± 0.05	2.57 ± 0.04	2.65 ± 0.08	2.70 ± 0.08	2.54 ± 0.02^b^
T_4_	2.38 ± 0.06	2.48 ± 0.03	2.55 ± 0.06	2.62 ± 0.06	2.66 ± 0.07	2.54 ± 0.01^b^
T_5_	2.35 ± 0.04	2.38 ± 0.07	2.46 ± 0.03	2.52 ± 0.07	2.59 ± 0.08	2.46 ± 0.06^c^
Means	2.36 ± 0.03^e^	2.46 ± 0.08^d^	2.54 ± 0.06^c^	2.61 ± 0.04^b^	2.68 ± 0.03^a^	–

T_o_ = control

T_BHA_ = synthetic antioxidant (butylated hydroxyl anisole)

T_1_ = cinnamon extract 0.5%

T_2_ = cinnamon extract 1%

T_3_ = cinnamon extract 1.5%

T_4_ = cinnamon extract 2%

T_5_ = cinnamon extract 2.5%

Means carrying same letters do not differ significantly

(*p* < 0.05)

**Table 3:** Effect of treatments and storage on free fatty acids content (%) of palm oil

Treatments	Storage (days)					Means
	0	7	14	21	28	
T_o_	0.497 ± 0.03	0.548 ± 0.02	0.599 ± 0.04	0.640 ± 0.05	0.721 ± 0.03	0.601 ± 0.05^a^
T_BHA_	0.498 ± 0.05	0.508 ± 0.01	0.518 ± 0.05	0.528 ± 0.03	0.559 ± 0.01	0.522 ± 0.02^e^
T_1_	0.477 ± 0.02	0.538 ± 0.04	0.588 ± 0.03	0.609 ± 0.05	0.690 ± 0.02	0.580 ± 0.07^b^
T_2_	0.487 ± 0.04	0.527 ± 0.01	0.568 ± 0.01	0.598 ± 0.03	0.679 ± 0.03	0.572 ± 0.03^b^
T_3_	0.483 ± 0.01	0.513 ± 0.03	0.553 ± 0.03	0.583 ± 0.01	0.666 ± 0.03	0.56 ± 00.07^c^
T_4_	0.503 ± 0.03	0.513 ± 0.06	0.533 ± 0.02	0.563 ± 0.02	0.646 ± 0.02	0.552 ± 0.03^c^
T_5_	0.493 ± 0.03	0.503 ± 0.05	0.523 ± 0.03	0.543 ± 0.03	0.616 ± 0.04	0.536 ± 0.05^d^
Means	0.491 ± 0.02^e^	0.521 ± 0.05^d^	0.555 ± 0.07^c^	0.580 ± 0.06^b^	0.654 ± 0.06^a^	–

T_o_ = control

T_BHA_ = synthetic antioxidant (butylated hydroxyl anisole)

T_1_ = cinnamon extract 0.5%

T_2_ = cinnamon extract 1%

T_3_ = cinnamon extract 1.5%

T_4_ = cinnamon extract 2%

T_5_ = cinnamon extract 2.5%

Means carrying same letters do not differ significantly

(*p* < 0.05)

The palm oil was also analysed periodically for TBA value during 28 days storage. The mean values obtained with respect to TBA value in fresh oil samples was 18.6 ± 0.73, whilst at 7, 14, 21 and 28 days the value increased gradually to 19.4 ± 1.45, 20.6 ± 1.56, 21.8 ± 1.56 and 24.7 ± 0.98. The means pertaining to treatments were observed as 22.4 ± 1.45, 20.1 ± 0.73, 21.8 ± 0.42, 21.2 ± 1.56, 20.7 ± 0.48, 20.5 ± 0.59, 20.2 ± 0.91, for T_o_, T_BHA_, T_1_, T_2_, T_3_, T_4_, T_5_, respectively (Table 4). Mean squares for Iodine value of palm oil showed non-significant effect of treatments on this trait. However, during storage this trait behaved differently. As depicted in Table 5., the means for Iodine value of respective treatments were 52.82 ± 2.12, 52.71 ± 2.38, 52.68 ± 2.96, 52.97 ± 2.14, 52.93 ± 2.12, 53.15 ± 2.38 and 52.71 ± 2.96 respectively, for T_o_, T_BHA_, T_1_, T_2_, T_3_, T_4_ and T_5_. Concerning the effect of storage, the data exhibited differences among storage days showing a little decline from 0 to 28th day. The values were 53.80 ± 2.12, 53.73 ± 2.96, 52.62 ± 2.63, 52.21 ± 2.12 and 51.90 ± 2.38 for 0, 7, 14, 21 and 28 days, correspondingly.

**Table 4:** Effect of treatments and storage on thiobarbituric acid content (ug/kg) of palm oil

Treatments	Storage (days)					Means
	0	7	14	21	28	
T _1_	18.6 ± 0.54	20.3 ± 0.48	22.1 ± 0.59	23.9 ± 0.42	26.9 ± 0.73	22.4 ± 1.45^a^
T_2_	18.8 ± 0.73	19.2 ± 0.98	20.0 ± 0.42	20.7 ± 1.56	21.7 ± 0.54	20.1 ± 0.73^f^
T_3_	18.5 ± 0.68	20.0 ± 1.56	21.4 ± 0.49	22.8 ± 0.67	26.3 ± 0.91	21.8 ± 0.42^b^
T _4_	18.3 ± 1.34	19.1 ± 0.59	20.5 ± 0.91	22.3 ± 1.45	26.1 ± 1.45	21.2 ± 1.56^c^
T_5_	18.3 ± 0.98	18.8 ± 1.56	20.1 ± 1.45	21.3 ± 0.91	24.2 ± 0.42	20.7 ± 0.48^d^
T_6_	18.6 ± 1.45	19.0 ± 0.54	20.0 ± 0.59	20.9 ± 0.54	24.2 ± 1.56	20.5 ± 0.59d^e^
T_7_	18.9 ± 0.91	19.4 ± 1.45	20.5 ± 0.73	20.5 ± 0.91	22.3 ± 1.45	20.2 ± 0.91e^f^
Means	18.6 ± 0.73^a^	19.4 ± 1.45^b^	20.6 ± 1.56^c^	21.8 ± 1.56^d^	24.7 ± 0.98^e^	–

T_o_ = control

T_BHA_ = synthetic antioxidant (butylated hydroxyl anisole)

T_1_ = cinnamon extract 0.5%

T_2_ = cinnamon extract 1%

T_3_ = cinnamon extract 1.5%

T_4_ = cinnamon extract 2%

T_5_ = cinnamon extract 2.5%

Means carrying same letters do not differ significantly

(*p* < 0.05)

**Table 5:** Effect of treatments and storage on Iodine value of palm oil

Treatments	Storage (days)					Means
	0	7	14	21	28	
T _1_	53.84 ± 2.63	53.68 ± 2.12	52.65 ± 2.96	52.09 ± 2.38	51.85 ± 2.14	52.82 ± 2.12^b^
T_2_	53.80 ± 2.96	53.55 ± 2.63	52.42 ± 2.38	52.05 ± 2.63	51.73 ± 2.96	52.71 ± 2.38^b^
T_3_	53.77 ± 2.12	53.53 ± 2.96	52.40 ± 2.38	52.02 ± 2.14	51.71 ± 2.63	52.68 ± 2.96^b^
T _4_	54.06 ± 2.38	53.82 ± 2.14	52.68 ± 2.12	52.30 ± 2.96	51.98 ± 2.12	52.97 ± 2.14^b^
T_5_	53.06 ± 2.96	54.03 ± 2.63	52.89 ± 2.63	52.51 ± 2.96	52.19 ± 2.63	52.93 ± 2.12^b^
T_6_	54.25 ± 2.12	54.00 ± 2.38	52.86 ± 2.12	52.48 ± 2.63	52.16 ± 2.14	53.15 ± 2.38^a^
T_7_	53.81 ± 2.14	53.53 ± 2.96	52.45 ± 2.14	52.04 ± 2.96	51.70 ± 2.96	52.71 ± 2.96^b^
Means	53.80 ± 2.12^a^	53.73 ± 2.96^a^	52.62 ± 2.63^b^	52.21 ± 2.12^b^	51.90 ± 2.38^c^	–

T_o_ = control

T_BHA_ = synthetic antioxidant (butylated hydroxyl anisole)

T_1_ = cinnamon extract 0.5%

T_2_ = cinnamon extract 1%

T_3_ = cinnamon extract 1.5%

T_4_ = cinnamon extract 2%

T_5_ = cinnamon extract 2.5%

Means carrying same letters do not differ significantly

(*p* < 0.05)

## Discussion

The reactive oxygen species may cause deterioration of lipid base foods during processing and storage. They produce off flavors and odors consequently reducing the shelf life and marketability of foods. In the past, synthetic antioxidants were extensively used to prevent lipid oxidation, however recently their use in food is governed by strict regulations owing to their detrimental effects on human health. Today consumers are more conscious about their health and demand food that is more like fresh, resultantly this leads to search for antioxidants from natural sources. In this connection, spices are lavish source of polyphenolic compounds that possess strong antioxidant capacity and can be potentially used to substitute the synthetic antioxidants from food products and confer some additional health effects. The results of the present study regarding TPC, DPPH and FRAP assay were in accordance with the finding of Thakur et al. [27]. They probed the antioxidant and antibacterial and activity of anti-inflammatory activity of ethanolic extract of cinnamon, turmeric and ginger. They observed that turmeric and ginger ethanolic extracts were effective against all tested bacteria. Furthermore, the FRAP test proved that the antioxidant potential of ginger was highest among all tested spices whilst the turmeric possess the lowest score. The antioxidant potential of Cinnamon is also good and the value of FRAP assay for cinnamon was 0.40 mM/100 g. They concluded that cinnamon extract has good antioxidant potential. Similar results were also recorded by Jayaprakasha et al. [17]. They studied that cinnamon extract also revealed the highest value for water extract followed by methanol, acetone and ethyl acetate. They concluded that cinnamon has good antioxidant activity and can be used as natural antioxidant in food products. Additionally, research conducted by Wang et al. [28], employed 45 essential oils to determine the antioxidant activity via total phenolics content and DPPH free radical scavenging activity. They noticed best antioxidant activity for leaf of cinnamon and clove bud oils. They narrated that ½ milliliter of these two oils has shown 96.74% and 96.12% DPPH ability, correspondingly. Next, for total phenolic content (mg/g of GAE) cinnamon, clove and thyme essential oils 1 mg/mL of each exhibited 420, 480, and 270, respectively. The present results are also in harmony with the findings of Su et al. [29]. They evaluated different spices for their radical scavenging capacity against DPPH, cation 2, 2-azinobis (3-ethylbenzothiazoline- 6-sulfonic acid) (ABTS +), peroxyl oxygen radical absorbance capacity (ORAC) and hydroxyl radicals. They noticed that the extracts of all tested spices including cinnamon exhibited appreciable antioxidant capacities. They found that the acetone extract of cinnamon showed the maximum ABTS + − scavenging ability and deduced that the spices especially cinnamon could be employed as a source of natural antioxidant for food products to improve human health and nutrition. The findings from the present study are also in line with the observations of Asimi et al. [30]. They conducted a study to evaluate the antioxidant activity and antimicrobial activities of five Indian spices including Cinnamon, Turmeric, Ginger, Cumin and Garlic. The antioxidant activity in decreasing order was observed as for DPPH Cumin > Garlic > Cinnamon > Turmeric > Ginger, for FRAP as Garlic > Cumin > Turmeric > Ginger > Cinnamon and for TPC as Turmeric > Cinnamon > Garlic > Cumin > Ginger. Hence their findings proved the antioxidant capacity of cinnamon and other spices. Storage conditions of refined deodorized palm oil (RDPO) have an effect on the quality and safety of the products [31, 32]. These results are in agreement with the previous findings of Umerie et al. [33]. They explored the efficacy of the use of *Ficus* exasperata leaves to improve the quality and stability of palm oil. They concluded that use of *Ficus* leaves enhanced the storage stability of palm oil. Liang et al. [34] determined the effect of synthetic and natural antioxidants on the oxidative stability of palm-based diesel and noticed that both antioxidants exposed beneficial effects in preventing the oxidation of palm diesel. Zhang et al. [35] explored the effect of natural and synthetic antioxidants on shelf life stability of sunflower oil. They investigated the peroxide values, *p*-anisidine value, free fatty acids, thiobarbituric acid-reactive substances and concluded that the natural antioxidants are better in reducing the oxidation damage to the oil as compared to synthetic one. The results of current research are in accordance with the findings of Zia-ur-Rehman et al. [36]. They explored the potential of natural antioxidants from potato peels in improving the oxidative stability of soybean oil and inferred that potato peel extract exhibited strong antioxidant capacity which is comparable to synthetic antioxidants BHA and BHT. Aleman et al. [37] concluded that garlic extract is a potent antioxidant and can be employed to reduce lipid oxidation in food products.

Both natural and synthetic antioxidants may inhibit or postpone the process of fat oxidation. An antioxidant can play important role in the prevention of oxidation as by acting as reducing agent, free radical scavenger, chelator and as singlet oxygen scavenger [38]. A number of different antioxidants are being registered as synthetic antioxidants yet few are acceptable as food additive by regulation due to toxicity and other allied harmful effects. Natural antioxidants are readily acceptable to modern consumers as being ideal food additives with good free radical scavenging activity moreover they are safer and healthier as compared to synthetic ones [24]. Cinnamon spice is one of the sources of effective antioxidants and enhances the efficacy of other important antioxidant molecules. It contains several potent antioxidants and is most important spice used for curtailing oxidative stress [39]. The antioxidant potential of cinnamon is attributed to array of flavonoid compounds that it contains. The essential oils present in cinnamon, including cinnamaldehyde, eugenol, and linalool, has been investigated in reference to peroxynitrite induced nitration and lipid peroxidation. Overall, research has proved that cinnamon exhibit higher antioxidant activity as compared to that of other dessert spices [18]. Recently, public’s fear about human health issues caused by food additives has aroused food scientists to keenness of trailing natural antioxidants from several sources. Until now the considerations on this matter is that natural antioxidants are predominantly plant phenolics arising different parts of plants. Some of the phenolic antioxidants from the plant source comprise flavonoids, coumarin, tocopherols, carotenoids, organic acids and cinnamic acid derivatives. The cinnamon extract scavenges harmful free radicals, which are implicated in common cancers and other degenerative diseases including poor brain activity [40]. Recent studies have observed the role of synthetic antioxidants as carcinogens [41]. Hence the search of effective, non-toxic, natural compounds with antioxidative ability has been strengthened in recent years [42].

Essential oils and eugenol from cinnamon bark has exhibited very strong in vitro antioxidant activities, reducing nitrotyrosine generation thus decreasing the peroxy nitrite prompted lipid oxidation [12]. The volatile oils obtained from cinnamon has revealed 55.9% and 66.9% antioxidant potential at 100 ppm and 200 ppm, accordingly [43]. Additionally, cinnamon bark extract was observed to be powerful in scavenging free radicals and ABTS radical cations, DPPH, hydroxyl & superoxides were also chelated by these compounds [44]. The current study supported by previous observations has proved that cinnamon extract has antioxidant activity related to its content of phenolic compounds and other antioxidants. It can be deduced that cinnamon can be employed as a source of natural antioxidant for food products to improve human health and nutrition.

### Limitations

Despite the proven antioxidant potential of the cinnamon extract further research is suggested to underpin recent analytical and biological evaluation approaches in order to gain a deeper understanding of possible antioxidant and health effects. Evaluation of cinnamon extract as antioxidant under different processing and storage conditions appears to be an appreciated future objective.

## Conclusions

The edible oils can be degraded through oxidation and decomposition of oxidation products, resulting in generation of alcohols, ketone, aldehyde, acids and hydrocarbons. They can badly affect the appearance, quality and palatability of a food product by creating obnoxious off-flavors and off-odors in food and hereafter affect safety, nutritional value, healthfulness, flavor, color and texture of different foods. These undesirable episodes of oxidation process can be inhibited by various ways including reduction of excess oxygen, and addition of oxidation inhibitors or antioxidants. There is growing trend for the use of natural antioxidants, for food ingredients preservation in recent years due to toxicity problems associated with synthetic antioxidants. The main purpose of the present research was to probe the antioxidant capacity of cinnamon spice. Moreover, the reducing ability of cinnamon extract on lipid oxidation was investigated after addition of extract in palm oil at different concentration levels along with synthetic antioxidant and control for comparison purposes. From the present study, it can be concluded that cinnamon extract has strong antioxidant potential and it can be successfully used in place of synthetic antioxidants in food preparations to improve human health and nutrition by reducing the production of hazardous oxidative derivates and free fatty acids.
